# Value of Magnifying Endoscopy With Narrow-Band Imaging and Confocal Laser Endomicroscopy in Detecting Gastric Cancerous Lesions

**DOI:** 10.1097/MD.0000000000001930

**Published:** 2015-11-06

**Authors:** Shuai Gong, Han-Bing Xue, Zhi-Zheng Ge, Jun Dai, Xiao-Bo Li, Yun-Jia Zhao, Yao Zhang, Yun-Jie Gao, Yan Song

**Affiliations:** From the Division of Gastroenterology and Hepatology, Ren Ji Hospital, School of Medicine, Shanghai Jiao Tong University; Shanghai Institute of Digestive Disease, Shanghai, China.

## Abstract

Although the respective potentials of magnifying endoscopy with narrow-band imaging (ME-NBI) and confocal laser endomicroscopy (CLE) in predicting gastric cancer has been well documented, there is a lack of studies in comparing the value and diagnostic strategy of these 2 modalities. Our primary aim is to investigate whether CLE is superior to ME-NBI for differentiation between gastric cancerous and noncancerous lesions. A secondary aim is to propose an applicable clinical strategy.

We conducted a diagnostic accuracy study involving patients with suspected gastric superficial cancerous lesions. White light endoscopy, ME-NBI, and CLE were performed diagnostic accuracy, sensitivity, specificity, positive predictive value, and negative predictive value between ME-NBI and CLE were assessed, as well as agreements between ME-NBI/CLE and histopathology.

This study involved 86 gastric lesions in 82 consecutive patients who underwent white light endoscopy, ME-NBI, and CLE before biopsy. The accuracy, sensitivity, and specificity for ME-NBI were 93.75%, 91.67%, and 95.45%, compared with 91.86%, 90%, and 93.48%, respectively, for CLE, for discrimination cancerous/noncancerous lesion (all *P* > 0.05). For undifferentiated/differentiated adenocarcinoma, CLE had a numerically but not statistically significantly higher accuracy than ME-NBI (81.25% vs 73.33%, *P* = 0.46). Agreements between ME-NBI/CLE and histopathology were near perfect (ME-NBI, κ = 0.87; CLE, κ = 0.84).

CLE is not superior to ME-NBI for discriminating gastric cancerous from noncancerous lesions. Endoscopist could make an optimal choice according to the specific indication and advantages of ME-NBI and CLE in daily practices.

## INTRODUCTION

Annually there are over 900,000 new cases of gastric cancer worldwide, representing a major burden on health services (gastric cancer remains the second leading cause of cancer deaths across the world).^1^ Screening for early gastric cancer (EGC) using multiple red-flag techniques can reduce the mortality rates.^2^ Precise endoscopic detection and biopsy of target lesion are crucial for diagnosis and management of EGC and precancerous lesions. However, routine biopsy sampling or endoscopic resection may increase the associated risks of bleeding and perforation. As for noncancerous lesions, multiple biopsies can be time-consuming and costly. In addition, local scars caused by previous biopsies make it difficult to perform subsequent examination or endoscopic resection.^3^ Thus in recent years there have been substantial interest in exploring new ways to diagnose gastrointestinal diseases real-time without biopsy.

These so-called “red-flag” techniques or new powerful digestive endoscopies offer an absolutely new visualization for gastrointestinal tract, including high-resolution and magnification endoscopy, computed virtual chromoendoscopy, confocal laser endomicroscopy (CLE), and endocytoscopy. ME-NBI (magnifying endoscopy with narrow-band imaging) is confirmed to have achieved excellent diagnostic accuracy in differentiating between noncancerous and cancerous lesions.^4–6^ CLE is the latest advanced endoscopic imaging technological modality. The first CLE platform was developed in 2004 which combined the application of confocal scanning microscope with conventional flexible endoscope.^7^ Subsequently, another system called probe-based CLE (pCLE), which conveys light with a fiber-optic probe bundled from a confocal microscope through the accessory channel of virtually any endoscope, was applied in clinical practice.^8^

Our center has demonstrated that ME-NBI could successfully distinguish between cancerous and noncancerous lesions and between undifferentiated and differentiated gastric adenocarcinomas, with both accuracies approximating 90%.^9^ Kakeji et al^10^ conducted an ex-vivo and in-vivo study on diagnosing gastric cancer using CLE, and concluded that CLE images correspond well with histopathological images. Zhang et al^11^ and Li et al^12^ then developed and refined CLE diagnostic criteria for gastric lesions especially in differentiating between normal and cancerous tissues, thus making it easy to apply in clinical practice with high validity and reliability. However, there are few studies in the diagnostic accuracy of ME-NBI compared to CLE for diagnosis of gastric cancerous lesions.

Consequently, we prospectively conducted this study to assess and compare the real-time diagnostic accuracy for gastric cancerous lesions between ME-NBI and CLE, and propose a practical clinical strategy, using histopathology as the “gold standard.”

## METHODS

### Participants

Participants were consecutively enrolled with suspected gastric superficial cancerous lesions previously diagnosed by conventional white light endoscopy (WLE) in either primary or secondary hospital, namely endoscopic findings corresponded with Paris endoscopic classification of superficial neoplastic lesions (elevated, flat, or depressed type) of the gastrointestinal tract.^13^ Exclusion criteria were as follows: patients with obvious advanced gastric cancer or previous gastrectomy; the patients’ condition was inadequate for receiving ME-NBI or CLE examination, such as those suffering liver cirrhosis, kidney dysfunction, acute gastrointestinal bleeding, esophageal and gastric varices, coagulopathy, or with known allergy to fluorescein sodium, etc.; patients with pregnancy, breastfeeding, younger than 18 year-old, or older than 80 years old; and those who were unable to sign informed consent.

All participants received informed consent before the study. The data collection was planned before endoscopic and histopathological examination at Ren Ji Hospital, School of Medicine, Shanghai Jiao Tong University. Local ethics committee approved the study protocol. The research was carried out in accordance with the Helsinki Declaration.

### Study Design

This study was conducted in accordance with Standards for Reporting of Diagnostic Accuracy initiative (STARD).^14,15^ We performed a randomization between ME-NBI and CLE endoscopic procedures to estimate the individual diagnostic value of each modality. Participants in group A had WLE followed by ME-NBI and CLE sequentially, while group B subjects had WLE, CLE, and ME-NBI in that sequence. All of these endoscopic procedures were performed by a single endoscopist (Han-Bing Xue) who was blind to patient's previous histopathological findings to avoid influence of varying experience of different endoscopists and previous diagnosis. We numbered patients according to the sequence of their examination date and carried out randomization via table of random number.

### Endoscopic Equipment and Procedure

Before the procedure, all patients have received deep sedation with intravenous propofol in combination with fentanyl, which was performed under continuous monitoring of vital signs with supplemental oxygen. All procedures were first performed by WLE involving the use of Olympus GIF-H260Z endoscope. During the examination, 30 mL of water with 20,000 units of pronase (Tide Pharmaceutical Co., Beijing, China) were used to wash the mucus and foam. When gastric lesions, especially superficial elevated, depressed and uneven lesions were discovered, ME-NBI or CLE should be performed to make a further examination.

ME-NBI was performed by using a magnifying endoscope (GIF-H260Z; Olympus, Tokyo, Japan). A transparent hood was attached to the tip of the endoscope to maintain the appropriate distance during the procedure. The CLE used in this study was an EG-3870CIK (Pentax, Tokyo, Japan). A contrast agent was injected intravenously (10% fluorescein sodium 5 mL, Wuzhou Pharmaceutical Co., Wuzhou, China). The sequence of these 2 equipments was randomized. All of above-mentioned procedures were performed by a single endoscopist (HBX) specialized in ME-NBI and CLE, and a real-time diagnosis of these 2 modalities were recorded by a research nurse. After examination of ME-NBI and CLE, forceps biopsy was obtained from the examined sites.

### Sample Size

ME-NBI had been reported to have a sensitivity of 92.9% to 97.3% for distinguishing gastric cancer from benign lesions, while the specificity was 84.4% to 96.8%.^6,9,16^ Although there have been few studies on CLE diagnosing gastric cancer, the valuable results thereof showed high reliability and validity (90.2%–92.6% for sensitivity, 97%–100% for specificity).^10,12,17^ We assumed that the sensitivity and specificity of ME-NBI versus CLE for detecting gastric cancerous lesions was 95% and 92%, 90% and 97%, respectively. The significance level of α was set at 0.05, and the allowable error of δ was set at 0.1, using the power of 80%. According to the sample size formula, the minimum sample size of cases required was 34 gastric cancerous lesions, and noncancerous cases should be at least 41.

### Diagnostic Criteria

The morphological class of lesions was recorded in accordance with the Paris Workshop guidelines.^13^ During this current study, we chose vessel plus surface (VS) classification as our diagnostic criteria of ME-NBI.^18^ In addition, if the lesions were diagnosed as cancerous by ”VS“ classification, then we would further divide them into differentiated and undifferentiated type according to the appearance of the fine network pattern and corkscrew pattern.^19^

As for CLE, we chose the gastric pit patterns classification and a simplified 2-tiered classification as diagnostic criteria.^11,12^ The simplified 2-tiered classification divides gastric lesions into noncancerous and cancer/high grade intraepithelial neoplasia (HGIN) lesions. The typical signs for cancer/HGIN lesions were as follows: irregular surface patterns, atypical glands, or disorganized patterns; irregular in shape and size and disordered dark cells; irregular, twisted, or unusual shaped microvessels. As for gastric cancerous lesions, type G1 (normal pits disappearing, with the appearance of diffusely atypical cells) and type G2 (normal pits disappearing, with appearance of typical cells) were responsible for undifferentiated and differentiated type, respectively. According to modified Vienna classification, high grade dysplasia (C 4.1) is regarded as mucosal high grade neoplasia and has a high risk of developing into intramucosal carcinoma or submucosal invasion; therefore, it should be given endoscopic or surgical local resection.^20^ In our study, we defined “cancerous lesion” as cancer or HGIN.

### Histopathological Assessment

All biopsy specimens obtained from examined sites were immediately fixed in 10% formalin, sectioned into 4 mm thick samples, and stained with hematoxylin-eosin (H&E) for routine histopathological analysis. Two experienced GI pathologists (XYC and YC) who were ignorant of the patients’ clinical history or previous endoscopic diagnosis reviewed all of the specimens independently. The histopathological criteria were based on the Updated Sydney System, the World Health Organization classification of digestive tumors and Vienna classification.^21–23^ If the lesion was diagnosed as adenocarcinoma, then it should be further divided into D-type (well or moderately differentiated adenocarcinoma or papillary adenocarcinoma) and UD-type (poorly differentiated adenocarcinoma, signet-ring cell carcinoma, or mucinous cell carcinoma). The histological analysis after surgical or endoscopic resection was accepted as final.

### Statistical Analysis

All statistical analyses were performed using SPSS 13.0 software package (SPSS Inc. Chicago, IL). Accuracy, sensitivity, specificity, positive predictive value (PPV), and negative predictive value (NPV) for ME-NBI and CLE were estimated with McNemar test, respectively, along with exact binomial 95% confidence intervals, with histopathology considered as the “gold standard”. χ^2^ test was used to compare rate and ratio. A 2-tailed *P* value <0.05 was considered statistically significant. Cohen kappa (κ) was used to represent agreement between ME-NBI/CLE and histopathology, with value of 0.01 to 0.20 indicating poor agreement, 0.21 to 0.40 fair, 0.41 to 0.60 moderate, 0.61 to 0.80 substantial, and 0.81 to 1.00 almost perfect.

## RESULTS

### Clinical Characteristics of Patients and Lesions

The study was performed from January 2013 to January 2014. A total of 86 consecutive patients were prospectively enrolled for study analysis. Four patients were withdrawn from the study before the randomization. Two patients were unable to receive deep sedation and the other 2 refused to have an injection of fluorescein sodium. Ultimately, 82 patients with 86 gastric lesions were analyzed. A total of 39 and 43 patients were assigned to group A and group B, respectively. There were 58 males and 24 females with a mean age of 59.3 ± 8.5 years (range from 29 to 79 years). The median size, macroscopic type, and histopathology were all summarized in Table 1.

**TABLE 1:**
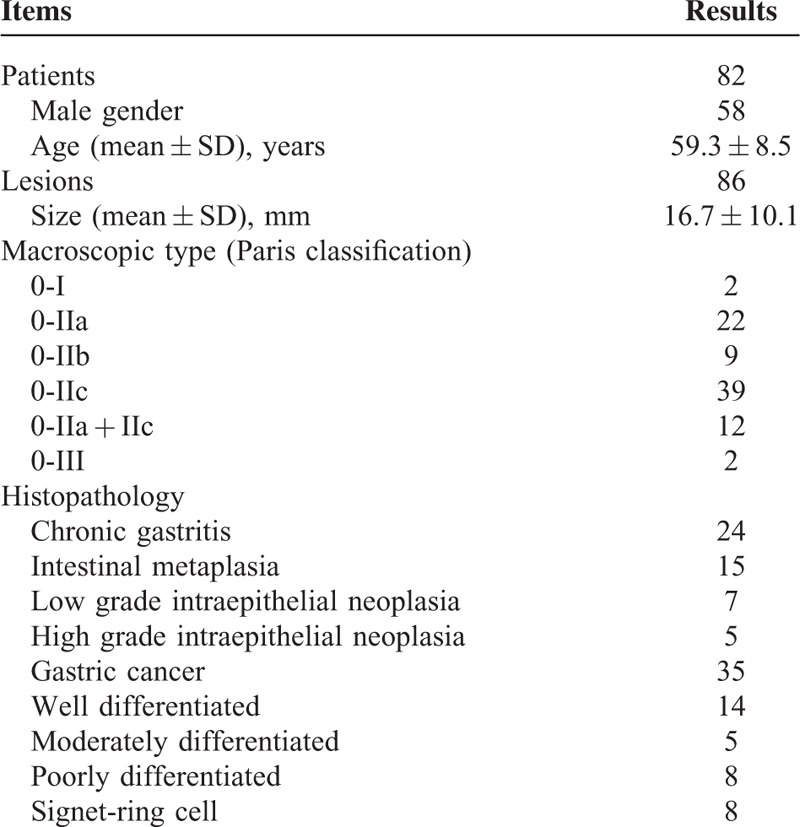
Patients’ Characteristics and Clinicopathological Features of Lesions

### Diagnostic Accuracy of ME-NBI and CLE for Gastric Cancerous Lesions

**FIGURE 1:**
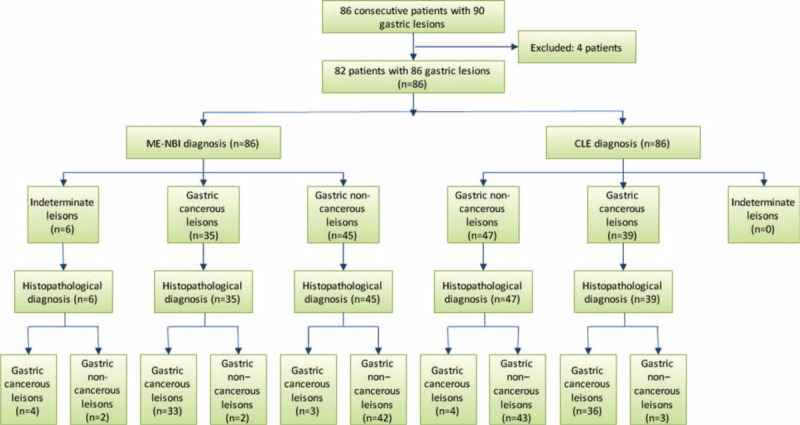
The flow diagram of a diagnostic accuracy. CLE = confocal laser endomicroscopy, ME-NBI = magnifying endoscopy with narrow-band imaging.

It took nearly 1 week to get histopathological diagnoses after examination of ME-NBI and CLE. No special treatment was given to participants except drug therapy for someone during this period and no adverse event was found. Figure 1 shows flow diagram of diagnostic accuracy in our study. Of the 86 gastric lesions, 40 (46.5%) were cancerous (gastric cancer/HGIN), while 46 (53.5%) were noncancerous (gastritis/intestinal metaplasia/low grade intraepithelial neoplasia). As for ME-NBI, endoscopist could not identify microvascular (MV) and microsurface (MS) pattern through dirty purulent surface in 6 ulcerative lesions, resulted in indeterminate diagnosis (4 cancerous and 2 noncancerous lesions). In contrast, the severity of total 86 lesions could be predicted by CLE in spite of the condition of lesion's surface. Table 2 displays the results of diagnostic value for differential diagnosis of gastric cancerous lesions using each imaging modality alone or in combination. Although the data of ME-NBI were relatively better than that of CLE, there was no statistical significance (the indeterminate lesions were not included in the final statistical phase for ME-NBI group). Among 80 lesions, 34 cancerous and 40 noncancerous lesions could be recognized with the combination of ME-NBI and CLE, the diagnostic sensitivity could reach at 94.44% (34/36), with the overall accuracy and specificity above 90%.

**TABLE 2:**
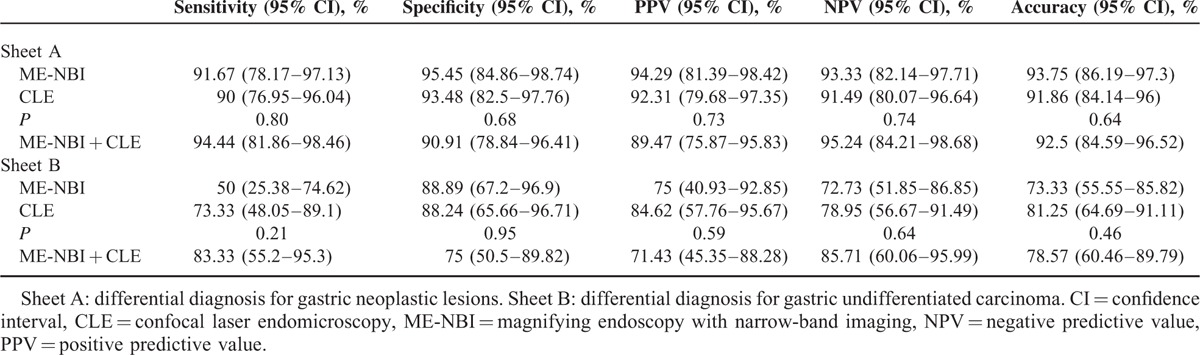
Comparison of Diagnostic Value of Magnifying Endoscopy With Narrow-Band Imaging and Confocal Laser Endomicroscopy for Gastric Lesions

### Differential Diagnosis for Subtypes of Gastric Cancer

**FIGURE 2:**
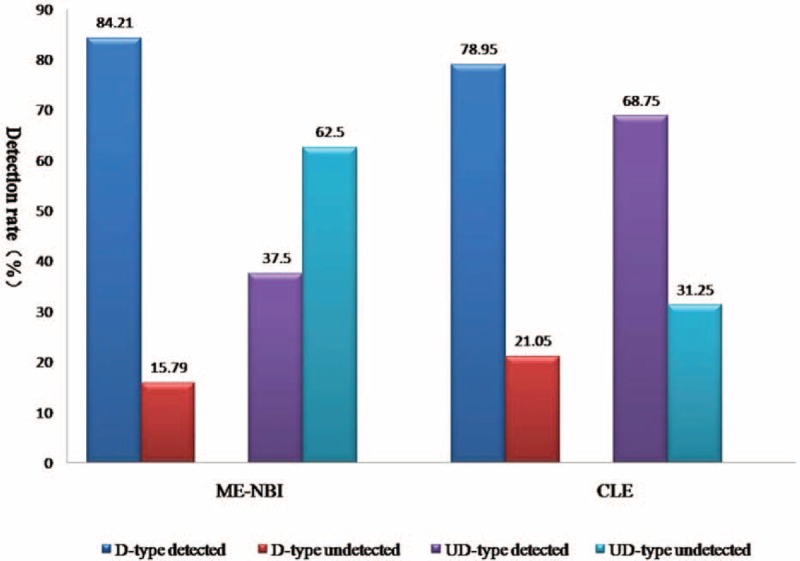
Detection rate for differentiated and undifferentiated type gastric carcinoma of magnifying endoscopy with narrow-band imaging and confocal laser endomicroscopy. CLE = confocal laser endomicroscopy, D-type = differentiated type gastric carcinoma, ME-NBI = magnifying endoscopy with narrow-band imaging, UD-type = undifferentiated type gastric carcinoma.

As showed in Table 1, of 35 gastric cancer, 14 were well differentiated, 5 were moderately differentiated, 8 were poorly differentiated, and 8 signet-ring cell. In terms of binary classification, 19 were differentiated (D-type), while 16 were undifferentiated (UD-type). CLE identified 32 gastric cancer (3 underestimated diagnosis), whereas 30 for ME-NBI (4 indeterminate and 1 underestimated diagnosis). The detection rate of D-type carcinoma was 84.21% (16/19) for ME-NBI and 78.95% (15/19) for CLE. Eleven of the 16 UD-type carcinomas (68.75%) were identified by CLE, in contrast, ME-NBI just identified 6 UD-type (37.5%) (Figure 2). Table 3 shows the comparisons between 2 imaging modalities and histopathological assessment for differential diagnosis of UD-type and D-type carcinomas. The sensitivity, accuracy, PPV, and NPV of CLE for distinguishing UD-type from D-type were numerically but not statistically significantly better than that of ME-NBI (Table 2).

**TABLE 3:**
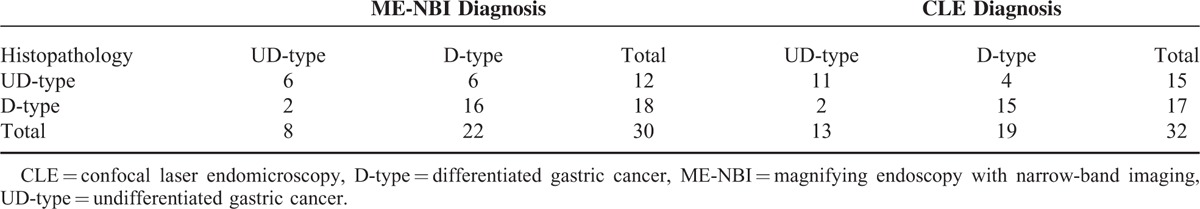
Comparison on Diagnosis of Undifferentiated Gastric Cancer by Magnifying Endoscopy With Narrow-Band Imaging and Confocal Laser Endomicroscopy

### Agreement Between ME-NBI/CLE and Histopathology

The agreements between new imaging modality and histopathology were all almost perfect with κ value of 0.87 for ME-NBI and 0.84 for CLE. Agreement remained perfect when combined the use of ME-NBI and CLE with a κ value of 0.85. The data also showed perfect agreement between ME-NBI and CLE, with a κ value of 0.82.

## DISCUSSION

Our study demonstrated CLE was not superior to ME-NBI for discriminating between gastric cancerous and noncancerous lesions with the sensitivity, specificity, and accuracy of 90%, 93.48%, and 91.86%, respectively. In the current study, the sensitivity was improved to 94.44% when ME-NBI combined with CLE, and the overall accuracy reached 92.5%. On the other hand, PPV of ME-NBI + CLE is worse than that of ME-NBI or CLE alone. That means, ME-NBI + CLE is helpful not to overlook cancerous lesion, but they will increase unnecessary therapy and take longer time. Although our primary single center study demonstrated the high validity and reliability of ME-NBI and CLE for gastric cancerous lesions, the high diagnostic accuracy and observer agreement were reported from limited experienced institute and lack of external validation from Western countries. It might be concerned with the different research areas between Eastern and Western countries. Owing to relatively different cancer prevalence, scholars from Western countries paid more attention to colorectal and esophageal cancers.

Unlike esophageal or colorectal cancers, no consensus on the criteria for the diagnosis of gastric cancer based on ME-NBI features has been established to date. In 2009, Yao et al reported an irregular MV pattern and/or an irregular MS pattern together with a clear demarcation line were the hallmarks of EGC.^18^ Their research group proposed the initial VS classification system. Recently, they conducted a multicenter prospective study to validate the VS classification system for screening EGC involving large samples (1092 patients). The accuracy, sensitivity, and specificity of high confidence ME-NBI diagnosis were 98.1%, 85.7%, and 99.4%, respectively.^24^ According to our experience, the observation of MS and MV patterns would be obstructed in lesions covered by dirty ulcerative things or fragile mucous membrane with a bleeding tendency. With regard to the current study, 6 of 86 lesions were indeterminate owing to invisible MS and MV patterns in ME-NBI group.

In our subgroup analysis, we paid attention to the differentiation of gastric cancer. According to Japanese gastric cancer treatment guidelines, D-type carcinoma has a lower rate of lymph node metastasis and a better prognosis than UD-type, thus resulting in different therapeutic strategy.^25^ Nakayoshi et al^19^ have described 2 characteristic patterns, one of which was called fine network pattern with an irregular MV network and the other was corkscrew pattern with isolated corkscrew-like vessels. They were confirmed to correlate with D-type and UD-type carcinoma, respectively. Another characteristic pattern proposed by Yokoyama for differentiated carcinoma was intra-lobular loop: irregular papillary MS, irregular MV pattern located in the gland duct.^26^ Some scholars believe that the MS structure of undifferentiated gastric cancer not invading the full layer of mucosa could still be mild dysplasia sometimes.^27^ For poorly differentiated adenocarcinoma and signet-ring cell carcinoma, mucosal surface may be covered by nonneoplastic foveolar epithelium, which shows the same color as the background mucosa at the early stage. It may be responsible for the low sensitivity in diagnosis of UD-type carcinoma using ME-NBI in our study.

**FIGURE 3:**
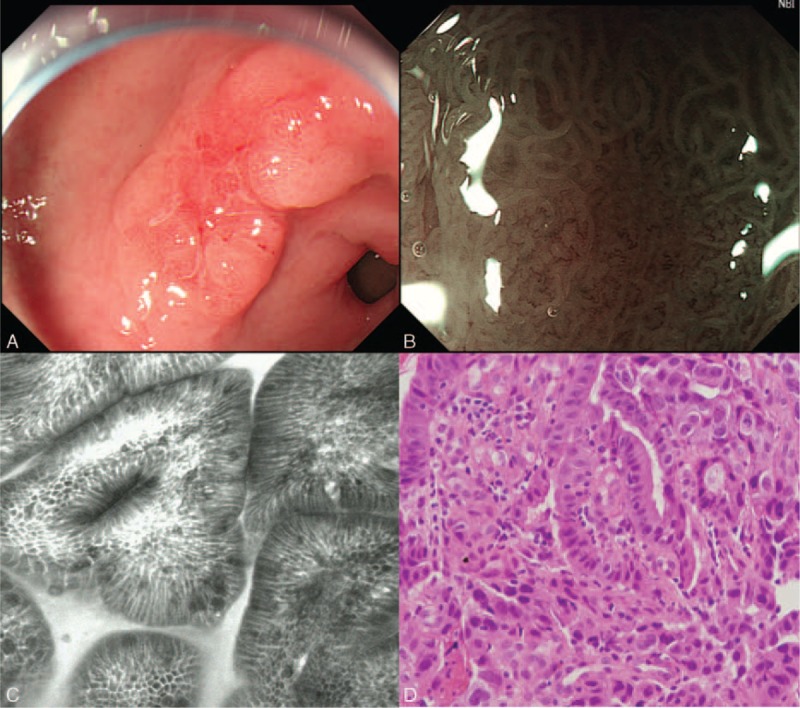
The characterizations of gastric well differentiated adenocarcinoma. (A) Image of white light endoscopy; (B) Image of magnifying endoscopy with narrow-band imaging; (C) Image of confocal laser endomicroscopy; (D) Histopathological image (H&E, ×400).

**FIGURE 4:**
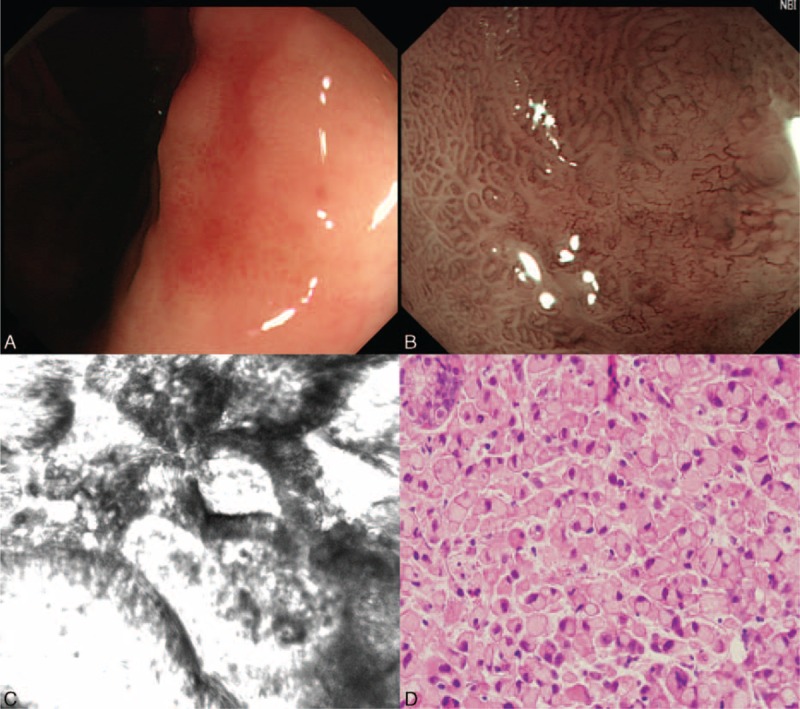
The characterizations of gastric signet-ring cell carcinoma. (A) Image of white-light endoscopy; (B) Image of magnifying endoscopy with narrow-band imaging; (C) Image of confocal laser endomicroscopy; (D) Histopathological image (H&E, ×400).

CLE is the latest advanced imaging device providing endoscopist a real-time in vivo histopathological images or optical biopsies of living cells and tissues during endoscopy. Asia, as a region of relatively higher gastric cancer prevalence than in the West, reports the most literatures on the utility of CLE in stomach. Nevertheless, the features of gastric cancer under CLE have not been standardized due to the difficulty in reading images which are totally different from either routine endoscopy or histopathology. Recent studies in China have described characteristic of gastric intraepithelial neoplasia and cancerous lesions. The sensitivity, specificity, and accuracy of real-time CLE for diagnosis of gastric superficial cancer or HGIN could be up to 88.9%, 99.3%, and 98.8%, respectively.^11,12,28^ In the present study, some endomicroscopic aspects for differential diagnosis between UD-type and D-type carcinoma were observed and summarized. D-type carcinoma appears to be hypervascular with vessels that were tortuous and dilated as well as irregular in shape and size, and the glands are atypical with irregular black cells (Figure 3). UD-type carcinoma usually exists with isolated, short-branched vessels, with almost destroyed or poorly arranged glands and irregular black cells (Figure 4). Our research also described the typical feature of gastric signet-ring cell on CLE: the presence of large vacuolated cells rich in mucin with solitary peripheral nuclei (typical signet-ring cells).^29^ As above-mentioned, the current study implied a phenomenon and trend that CLE might has advantage than ME-NBI for the diagnosis of those early gastric UD-type adenocarcinoma with nonneoplastic foveolar epithelium mucosal surface. However, there was no statistical significance between CLE and ME-NBI in discriminating between UD-type and D-type gastric adenocarcinoma (accuracy, 81.25% vs 73.33%; *P* = 0.46). Multicenter studies with larger sample sizes are crucial for confirming this trend in the future.

Some researchers suggest CLE was superior to NBI on gastric intestinal metaplasia and intraepithelial neoplasia.^30,31^ Our study showed CLE was not superior to ME-NBI for differentiation between gastric cancerous and noncancerous lesions. The reasons for this outcome might be that our center had richer experience on ME-NBI than CLE, and we withdraw 6 indeterminate cases for final statistical analysis, thus increasing diagnostic validity in ME-NBI group. The high PPV (94.29% and 92.31%), NPV (93.33% and 91.49%), and agreement with histopathology (0.87 and 0.84) of ME-NBI and CLE confirmed the enormous significance of these 2 modalities in clinical practice. Clinician could make an appropriate and quick judgment based on either single or combined use of them. If the lesion is suspected to be cancerous, then a target or multiple biopsies should be performed to confirm the histopathology. On the other hand, when the lesion is considered noncancerous by experienced endoscopist using ME-NBI or CLE, a negative biopsy could be avoided.

**FIGURE 5:**
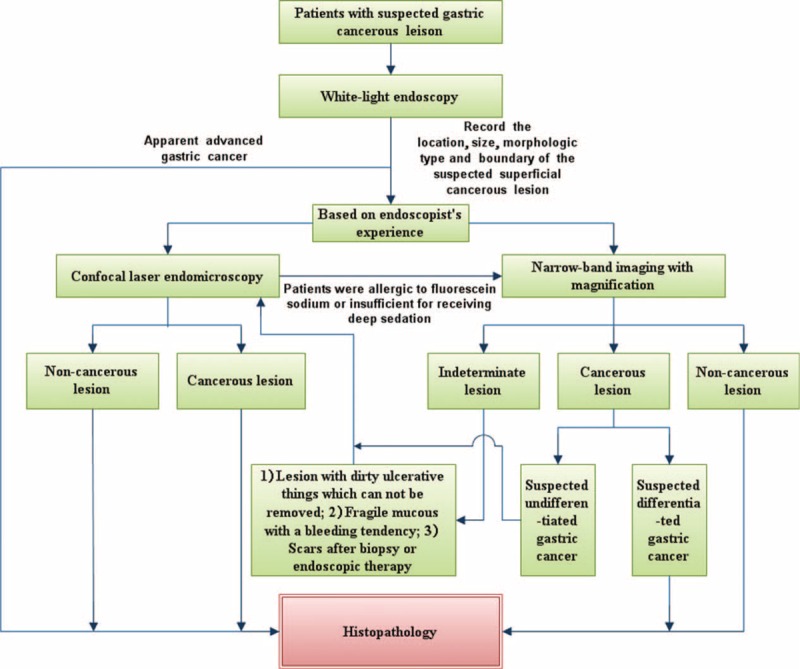
A clinical strategy for diagnosis of gastric cancerous lesions.

The secondary aim of the study was to propose an applicable clinical strategy for diagnosing gastric cancerous lesions. The current Asia-Pacific consensus does not recommend NBI as the initial modality for the detection of EGC during screening endoscopy. However, they suggest that NBI can be used in differentiation of neoplastic from nonneoplastic lesions, as well as distinguishing tumor margins from nonneoplastic surrounding mucosa.^32^ There has been no consensus published on diagnosis of gastric cancer by CLE. In our opinion, CLE is not suitable for screening EGC due to the difficulty in surveying the whole gastric lumen with limited field of vision. We recommend conventional WLE in screening endoscopic examination, as soon as gastric superficial lesions have been identified. ME-NBI or CLE could be applied in further examination according to endoscopist's experience. CLE is preferred if: surface of lesion is covered by thick and dirty ulcerative things, and MS and MV patterns could not be observed; fragile mucous membrane with a bleeding tendency; fibrosis after biopsy or endoscopic surgery; and suspicious undifferentiated gastric carcinoma. If the patient is insufficient to receive deep intravenous sedation or allergic to fluorescein sodium, ME-NBI would be an appropriate choice. Histopathological examination remained “gold standard” for final diagnosis. A clinical strategy for diagnosis of gastric cancerous lesions can now be proposed based on advantages of each modality mentioned above (Figure 5).

The current study has several limitations that should be noted. First, we only enrolled patients suspected with gastric cancerous lesions that have been diagnosed previously, thus raising the proportion of cancerous lesions. In addition, patients with obviously advanced gastric cancer were excluded. Second, the subjective experience of the endoscopists in ME-NBI and CLE is varied. Namely, the bias of endoscopist herself could affect the diagnostic accuracy. The standardized diagnosis criteria and learning curve of each modality are crucial. To avoid the influence of varying experience of different endoscopists, we chose a single endoscopist to perform examinations. However, since the single endoscopist was unable to be blind to previous findings among ME-NBI and CLE observation, it might improve diagnostic accuracy of the later one. So, we made a randomization between the order of 2 devices, this intervention could balance the validity of each modality, nevertheless, enhanced the real diagnostic accuracy of ME-NBI and CLE. As is known to all, NBI was perhaps hampered by mucous and blood due to endoscope contact on the mucosa during NBI or CLE. The fact demonstrated an advantage of CLE, but also a possibility that the tandem randomized comparison was unfair to compare the 2 modalities. Third, the number of gastric cancerous lesions is relatively small, though it meets the required sample size, especially when comparing the diagnostic yield between UD-type and D-type gastric adenocarcinoma. Finally, this was a single-center study where cost-effectiveness of ME-NBI and CLE is not evaluated. Multicenter randomized controlled prospective studies with larger sample sizes are necessary.

In conclusion, gastric cancerous lesions could be detected by ME-NBI or CLE with high validity and reliability. CLE is not superior to ME-NBI for discriminating gastric cancerous from noncancerous lesions. Endoscopist could make an optimal choice according to the specific indication and advantages of ME-NBI and CLE in daily practices. The unified diagnostic strategy and consensus on the criteria of ME-NBI and CLE for the diagnosis of gastric cancerous lesions are expected to be proposed in the future.
